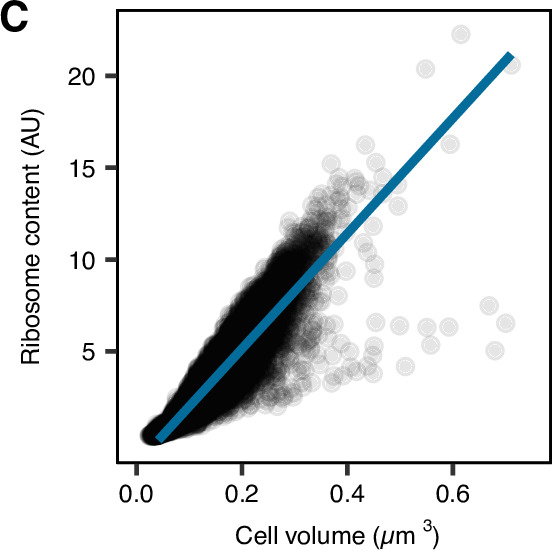# Erratum for Brüwer et al., “*In situ* cell division and mortality rates of SAR11, SAR86, *Bacteroidetes*, and *Aurantivirga* during phytoplankton blooms reveal differences in population controls”

**DOI:** 10.1128/msystems.01196-23

**Published:** 2023-12-20

**Authors:** Jan D. Brüwer, Luis H. Orellana, Chandni Sidhu, Helena C. L. Klip, Cédric L. Meunier, Maarten Boersma, Karen H. Wiltshire, Rudolf Amann, Bernhard M. Fuchs

## ERRATUM

Volume 8, no. 3, e01287-22, 2023, https://doi.org/10.1128/msystems.01287-22. During the publication process, Fig. 2 was corrupted and raw data points in panel C were omitted from the HTML version, although they do appear in the PDF version. The panel should appear as shown below.

**Fig 2:**