# Supplementary material for: In situ cell division and mortality rates of SAR11, SAR86, Bacteroidetes, and Aurantivirga during phytoplankton blooms reveal differences in population controls
Source: mSystems. 2023 May 17;8(3):e01287-22. doi: 10.1128/msystems.01287-22 (PMC10308942; doi:10.1128/msystems.01287-22)
Supplement: FIG S1 — Total DAPI-stained cell counts with chlorophyll a concentration (grey, background), temperature, and photosynthetically active radiation (PAR) during the spring phytoplankton bloom (A) 2018 and (B) 2020. [file msystems.01287-22-s0001.pdf]

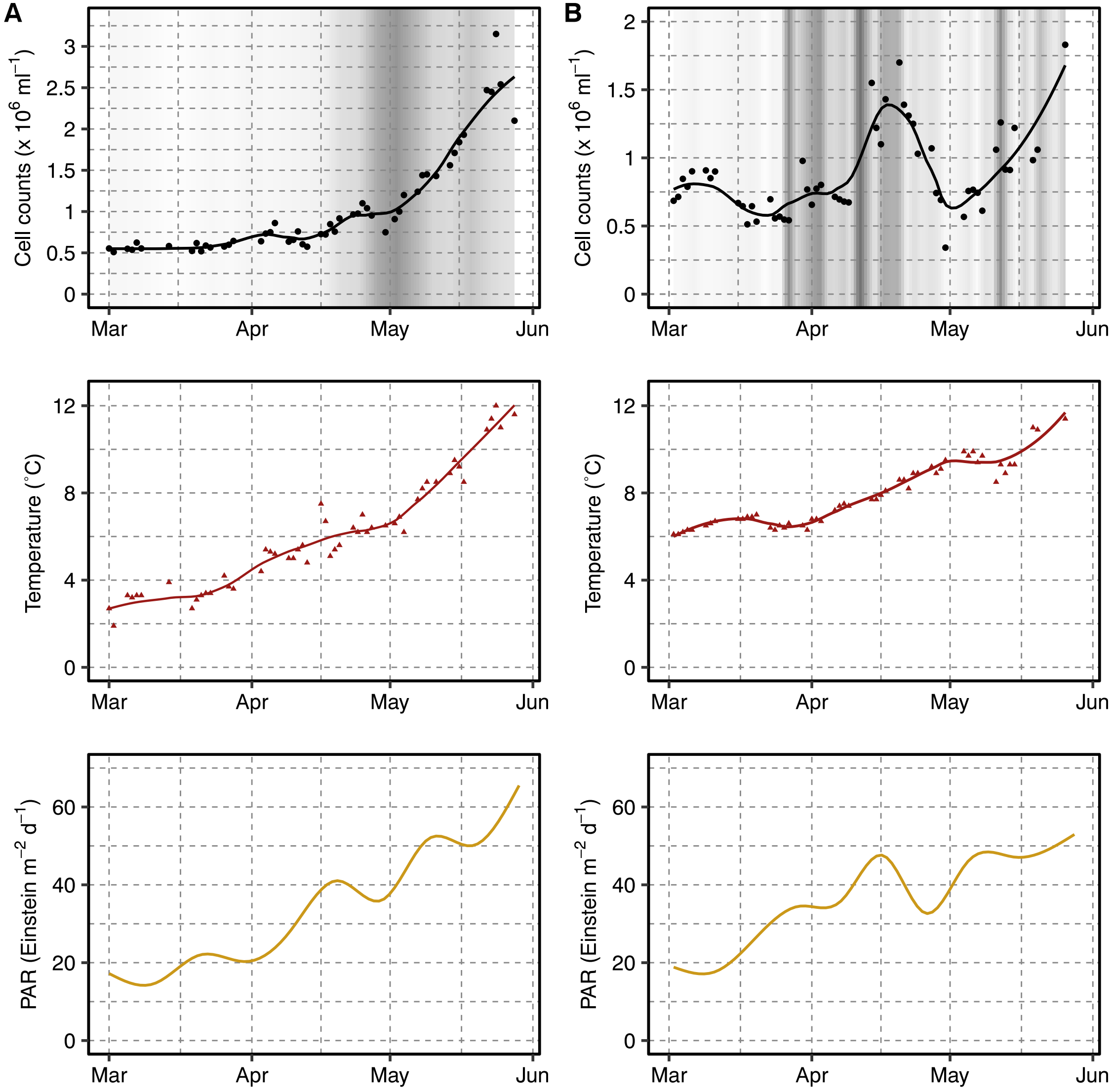

Figure S1 Total DAPI-stained cell counts with chlorophyll a concentration (grey, background), temperature, and photosynthetically active radiation (PAR) during the spring phytoplankton bloom (A) 2018 and (B) 2020.
