# Supplementary material for: In situ cell division and mortality rates of SAR11, SAR86, Bacteroidetes, and Aurantivirga during phytoplankton blooms reveal differences in population controls
Source: mSystems. 2023 May 17;8(3):e01287-22. doi: 10.1128/msystems.01287-22 (PMC10308942; doi:10.1128/msystems.01287-22)
Supplement: FIG S2 — Taxon and year-specific correlation of cellular parameters measured with FISH. (A) Ribosome content to cell volume. (B) Ribosome content over FDC. (C) Cell volume over FDC. Box-whisker plots in (B) and (C) range from 25th to 75th percentile and the whiskers represent 1.5* interquartile range. Outliers are visualized by dots. Mean is drawn as a solid line inside the boxes. Statistic results of regressions are reported supplementary results. [file msystems.01287-22-s0002.pdf]

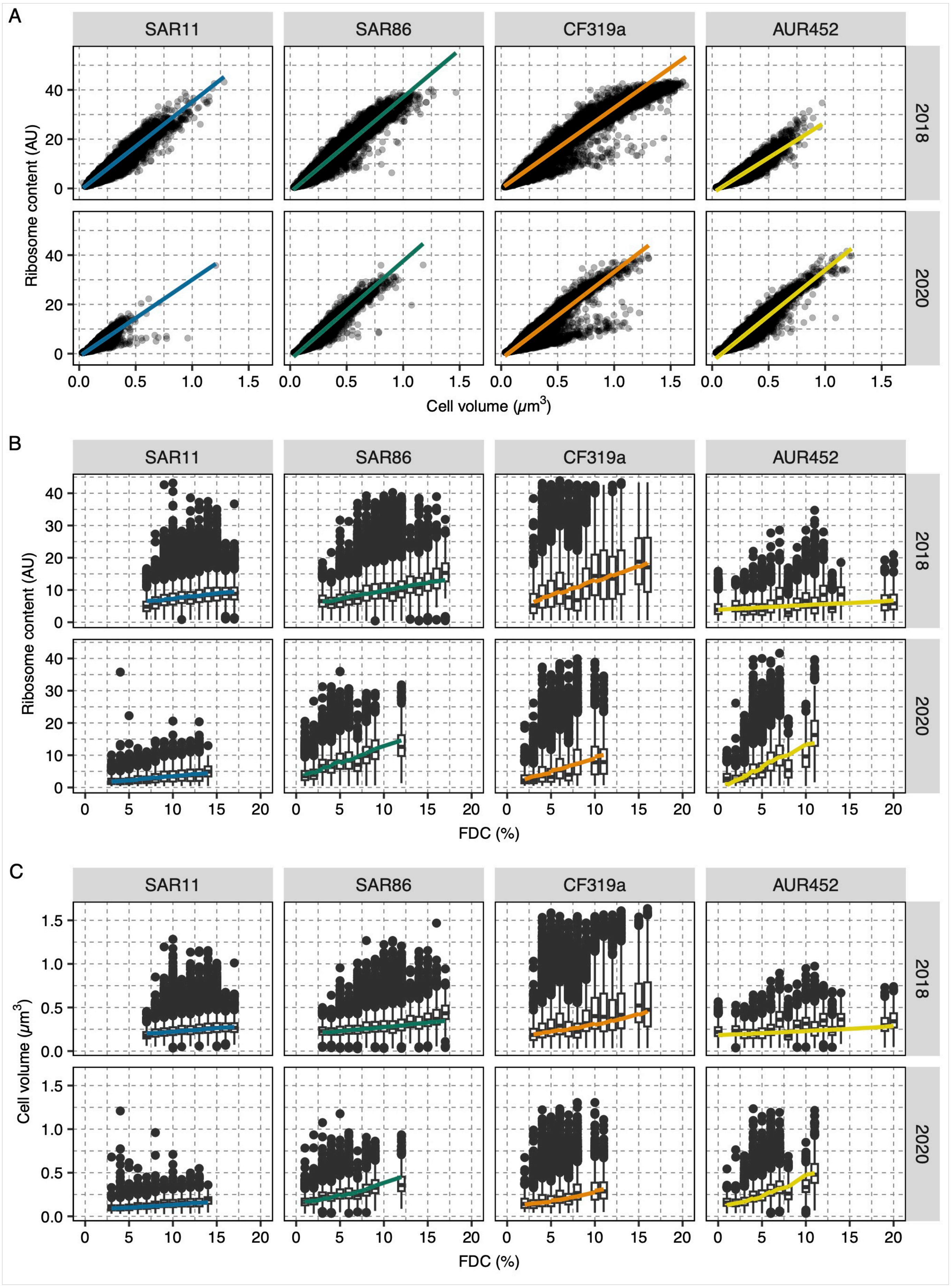

Figure S2 Taxon and year-specific correlation of cellular parameters measured with FISH. (A) Ribosome content to cell volume. (B) Ribosome content over FDC. (C) Cell volume over FDC. Box-whisker plots in (B) and (C) range from 25th to 75th percentile and the whiskers represent 1.5* interquartile range. Outliers are visualized by dots. Mean is drawn as a solid line inside the boxes. Statistic results of regressions are reported supplementary results.
