# Supplementary material for: In situ cell division and mortality rates of SAR11, SAR86, Bacteroidetes, and Aurantivirga during phytoplankton blooms reveal differences in population controls
Source: mSystems. 2023 May 17;8(3):e01287-22. doi: 10.1128/msystems.01287-22 (PMC10308942; doi:10.1128/msystems.01287-22)
Supplement: FIG S3 — (A) Linear correlations of microscopically-derived FDC and cell division rates determined by dilution experiments. Information about the linear regression can be found in table S2 (at doi.org/10.6084/m9.figshare.22290166). (B) Taxon-specific grazing rates over mortality rates in 2020. Taxon-specific grazing rates were determined with dilution experiments on 5 time-points during the 2020 phytoplankton bloom. Mortality rates were calculated from cell division rates and on net growth. Net growth rates could not be retrieved for SAR11 and SAR86 on one sampling day, as local regressions of abundance values were computed to calculate net growth and the regressions were partly negative. Black line is an ideal line of 1:1 correlation of grazing and mortality rates. [file msystems.01287-22-s0003.pdf]

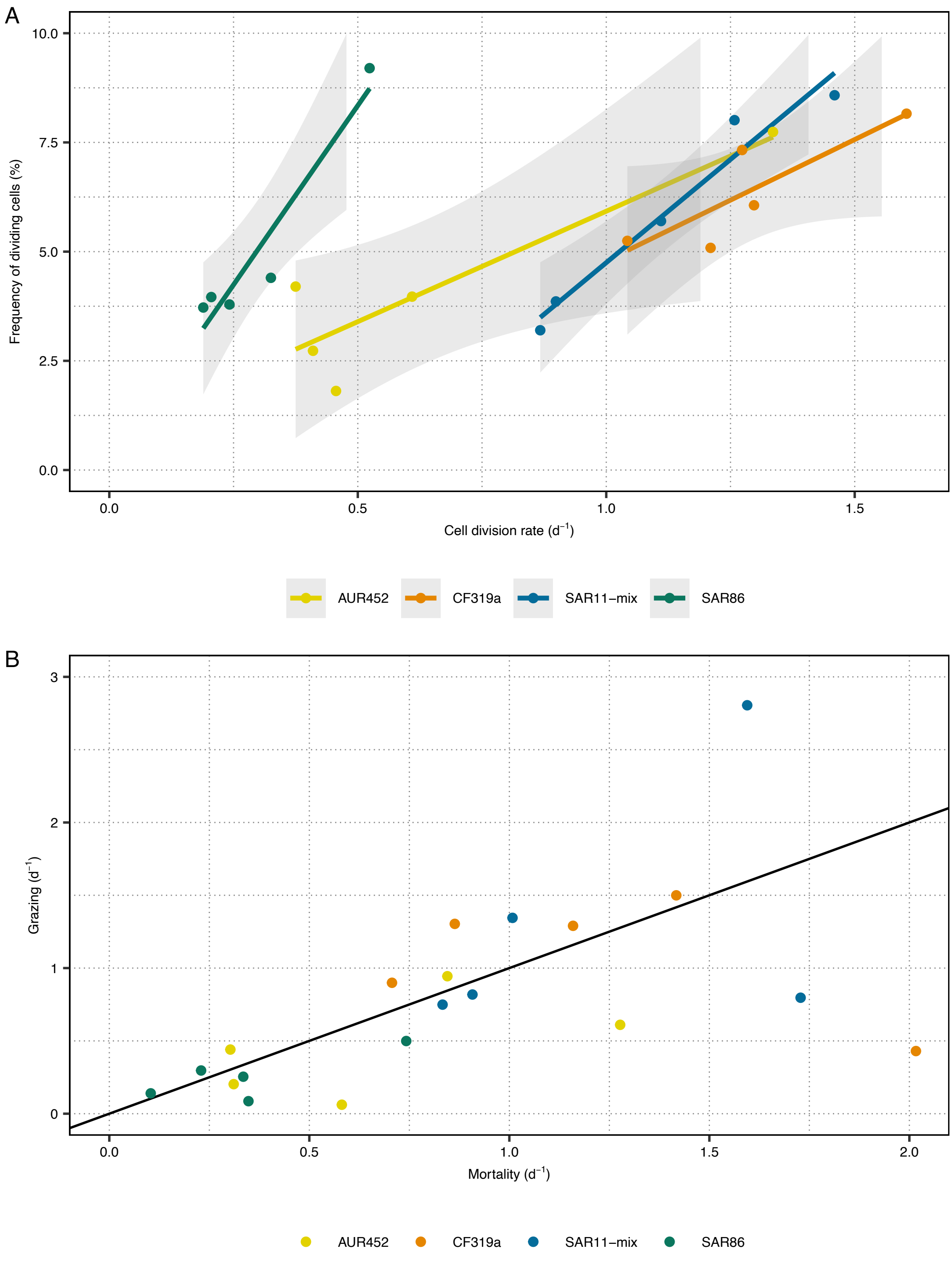

Figure S3 (A) Linear correlations of microscopically-derived FDC and cell division rates determined by dilution experiments. Information about the linear regression can be found in table S2 (at doi.org/10.6084/m9.figshare.22290166). (B) Taxon-specific grazing rates over mortality rates in 2020. Taxon-specific grazing rates were determined with dilution experiments on 5 time-points during the 2020 phytoplankton bloom. Mortality rates were calculated from cell division rates and on net growth. Net growth rates could not be retrieved for SAR11 and SAR86 on one sampling day, as local regressions of abundance values were computed to calculate net growth and the regressions were partly negative. Black line is an ideal line of 1:1 correlation of grazing and mortality rates.
