# Supplementary material for: In situ cell division and mortality rates of SAR11, SAR86, Bacteroidetes, and Aurantivirga during phytoplankton blooms reveal differences in population controls
Source: mSystems. 2023 May 17;8(3):e01287-22. doi: 10.1128/msystems.01287-22 (PMC10308942; doi:10.1128/msystems.01287-22)
Supplement: FIG S4 — Phylogenetic tree with SAR11 MAGs that have >50% completeness and <5% contamination described in this study with previously published SAR11 single amplified genomes (SAGs) (Haro‐Moreno JM, Rodriguez‐Valera F, Rosselli R, Martinez‐Hernandez F, Roda‐Garcia JJ, Gomez ML, Fornas O, Martinez‐Garcia M, López‐Pérez M. 2020. Environmental Microbiology 22:1748-1763) and SAR11 isolates (Delmont TO, Kiefl E, Kilinc O, Esen OC, Uysal I, Rappe MS, Giovannoni S, Eren AM. 2019. eLife 8:e46497). Colours according to clade assignment (indicated in outer ring) according to the literature (see above). [file msystems.01287-22-s0004.pdf]

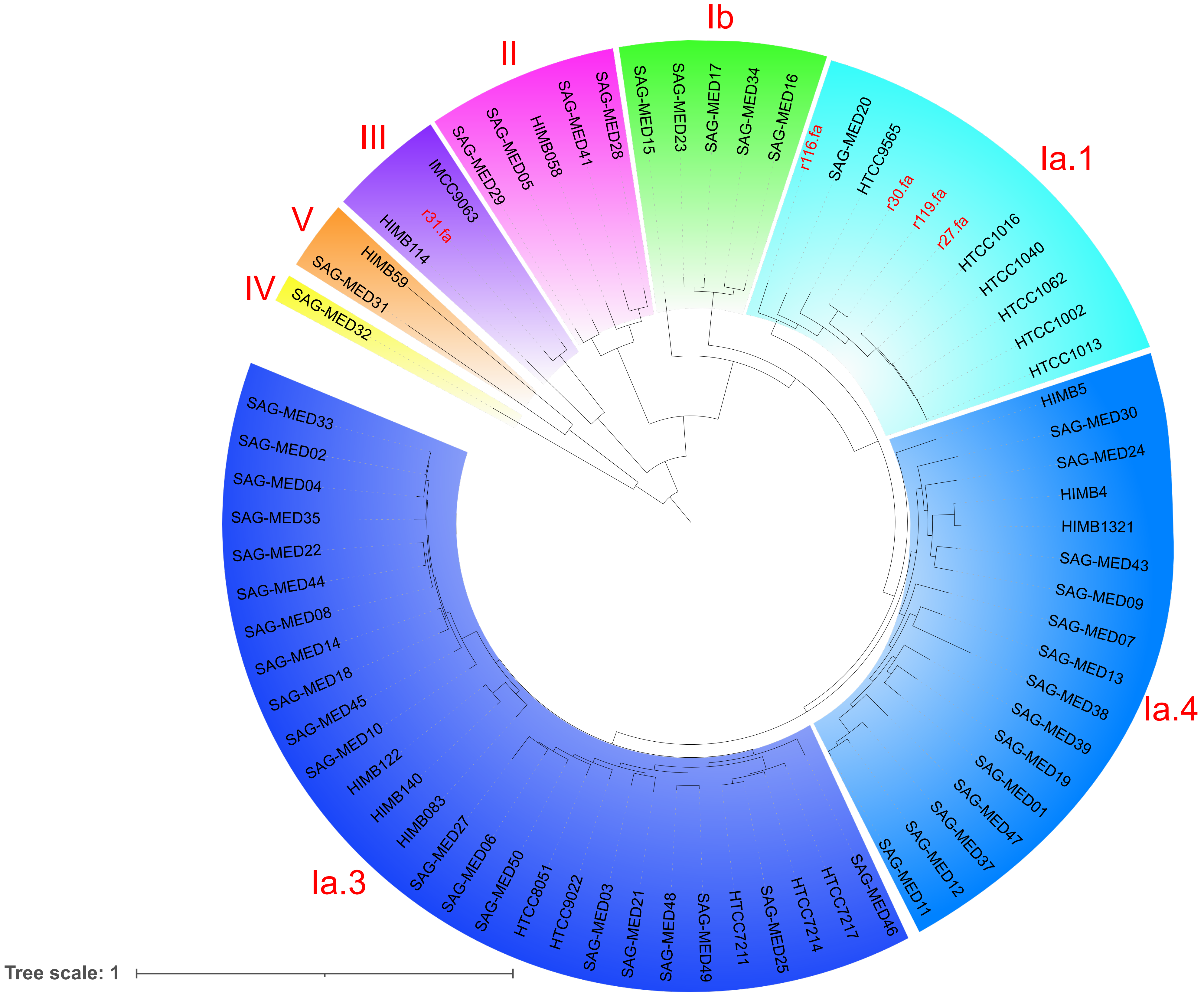

Figure S4 Phylogenetic tree with SAR11 MAGs that have >50% completeness and <5% contamination described in this study with previously published SAR11 single amplified genomes (SAGs) (Haro-Moreno JM, Rodriguez-Valera F, Rosselli R, Martinez-Hernandez F, Roda-Garcia JJ, Gomez ML, Fornas O, Martinez-Garcia M, López-Pérez M. 2020. Environmental Microbiology 22:1748-1763) and SAR11 isolates (Delmont TO, Kiefl E, Kilinc O, Esen OC, Uysal I, Rappe MS, Giovannoni S, Eren AM. 2019. eLife 8:e46497). Colours according to clade assignment (indicated in outer ring) according to the literature (see above).
