# Supplementary material for: In situ cell division and mortality rates of SAR11, SAR86, Bacteroidetes, and Aurantivirga during phytoplankton blooms reveal differences in population controls
Source: mSystems. 2023 May 17;8(3):e01287-22. doi: 10.1128/msystems.01287-22 (PMC10308942; doi:10.1128/msystems.01287-22)
Supplement: FIG S5 — Metagenome-based assessment of microbial growth in 2018. Relative abundances of metagenome assembled genomes (MAGs; A-D), as well as GRiD values (E–H), of all four taxa were calculated across the spring bloom 2018. For SAR11, SAR86, and Aurantivirga results of individual MAGs are visualized. For Bacteroidetes, results are summarized on the genus level (C, G with standard deviation). (I-L) MAG-derived GRiD values plotted versus FDC for phytoplankton spring bloom 2018. [file msystems.01287-22-s0005.pdf]

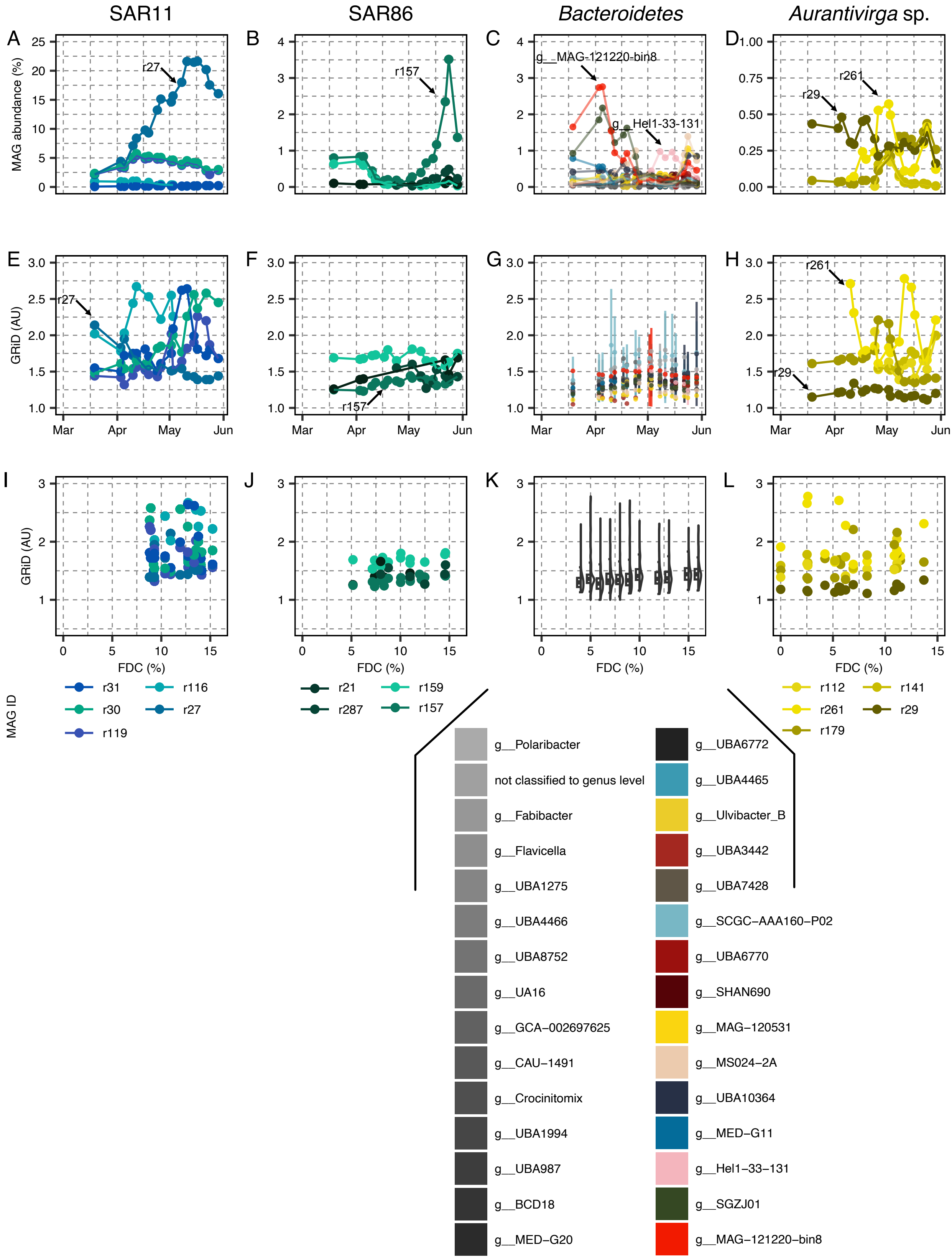

Figure S5 *Metagenome-based assessment of microbial growth in 2018*. Relative abundances of metagenome assembled genomes (MAGs; A-D), as well as GRiD values (E–H), of all four taxa were calculated across the spring bloom 2018. For SAR11, SAR86, and *Aurantivirga* results of individual MAGs are visualized. For *Bacteroidetes*, results are summarized on the genus level (C, G with standard deviation). (I-L) MAG-derived GRiD values plotted versus FDC for phytoplankton spring bloom 2018.
