# Supplementary material for: In situ cell division and mortality rates of SAR11, SAR86, Bacteroidetes, and Aurantivirga during phytoplankton blooms reveal differences in population controls
Source: mSystems. 2023 May 17;8(3):e01287-22. doi: 10.1128/msystems.01287-22 (PMC10308942; doi:10.1128/msystems.01287-22)
Supplement: FIG S6 — Comparison of GRiD values and abundance estimates from the 2018 phytoplankton spring bloom with different mappings for (A) SAR11, (B) SAR86, and (C) Aurantivirga. From left to right: GRiD values from customized alignment, retrieved with BBmap; GRiD software in default mode (minimum coverage: 5); GRiD software in default mode (minimum coverage: 5) and re-alignment of ambiguous reads with Pathoscope2 within the GRiD software. [file msystems.01287-22-s0006.pdf]

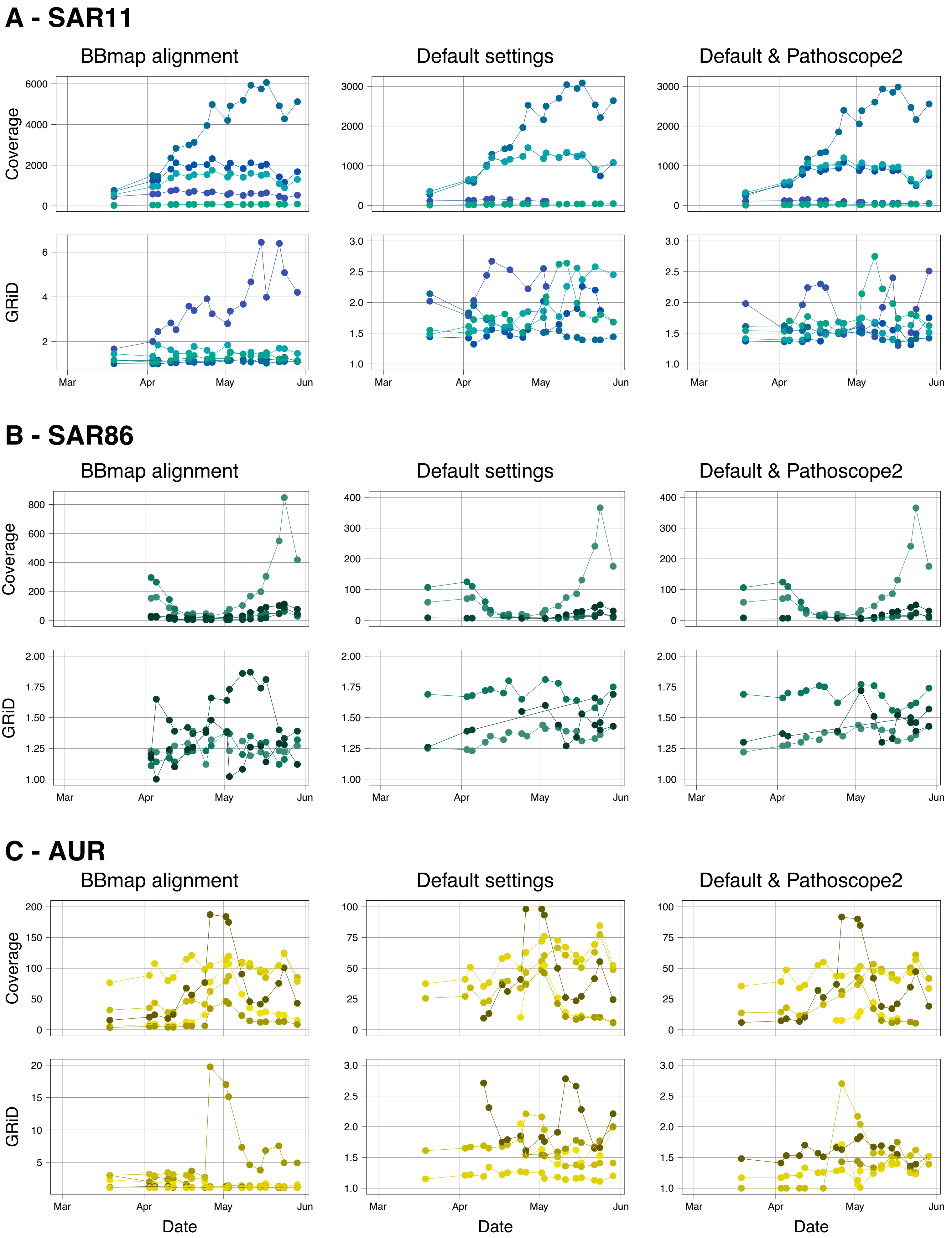

Figure S6 Comparison of GRiD values and abundance estimates from the 2018 phytoplankton spring bloom with different mappings for (A) SAR11, (B) SAR86, and (C) *Aurantivirga*. From left to right: GRiD values from customized alignment, retrieved with BBmap; GRiD software in default mode (minimum coverage: 5); GRiD software in default mode (minimum coverage: 5) and re-alignment of ambiguous reads with Pathoscope2 within the GRiD software.
