# Supplementary material for: In situ cell division and mortality rates of SAR11, SAR86, Bacteroidetes, and Aurantivirga during phytoplankton blooms reveal differences in population controls
Source: mSystems. 2023 May 17;8(3):e01287-22. doi: 10.1128/msystems.01287-22 (PMC10308942; doi:10.1128/msystems.01287-22)
Supplement: FIG S7 — Relationship of SAR11 FDC to PAR for 2018 and 2020. Left: FDC over Photosynthetically active radiation (PAR) on the left. A loess moving average is plotted. Right: PAR (ochre) and FDC (black) are plotted over the spring blooms with chlorophyll a plotted in the background. Scale is the same as Fig. 1 and 2. Red dashed line indicate potential threshold of 25 Einstein m-2 d–1 for SAR11 activity in the beginning of the bloom. [file msystems.01287-22-s0007.pdf]

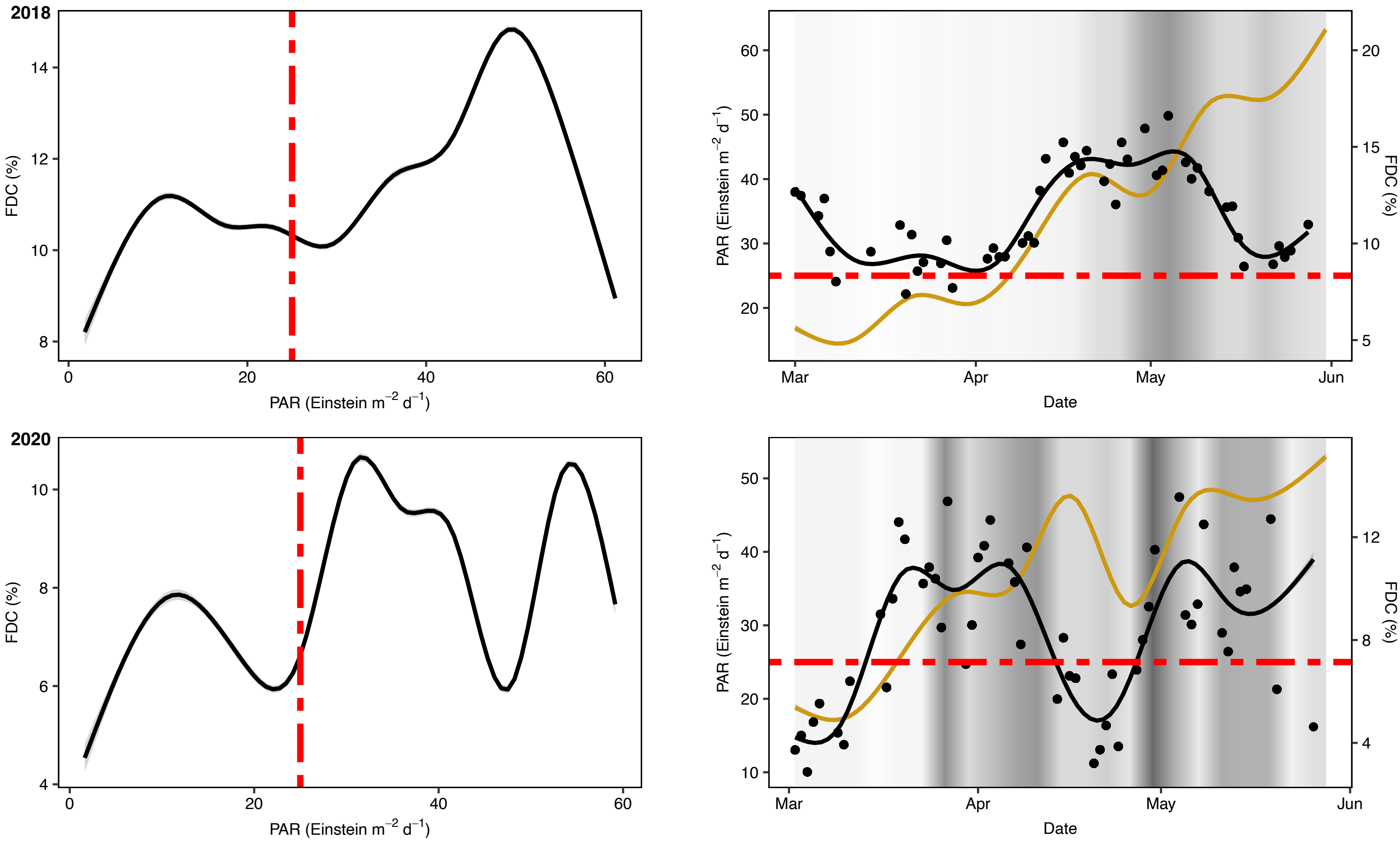

Figure S7 Relationship of SAR11 FDC to PAR for 2018 and 2020. Left: FDC over Photosynthetically active radiation (PAR) on the left. A *loess* moving average is plotted. Right: PAR (ochre) and FDC (black) are plotted over the spring blooms with chlorophyll a plotted in the background. Scale is the same as Fig. 1 and 2. Red dashed line indicate potential threshold of 25 Einstein m^{-2} d^{-1} for SAR11 activity in the beginning of the bloom.
