# Supplementary material for: In situ cell division and mortality rates of SAR11, SAR86, Bacteroidetes, and Aurantivirga during phytoplankton blooms reveal differences in population controls
Source: mSystems. 2023 May 17;8(3):e01287-22. doi: 10.1128/msystems.01287-22 (PMC10308942; doi:10.1128/msystems.01287-22)
Supplement: FIG S8 — Correlation of CARD-FISH and tetra-labelled FISH cell volumes and signal intensities for Bacteroidetes and SAR11 cells from selected 2020 spring bloom dates. Displayed are means of each sampling day and linear regression of the means. The displayed statistics are for the linear regression model on the means. [file msystems.01287-22-s0008.pdf]

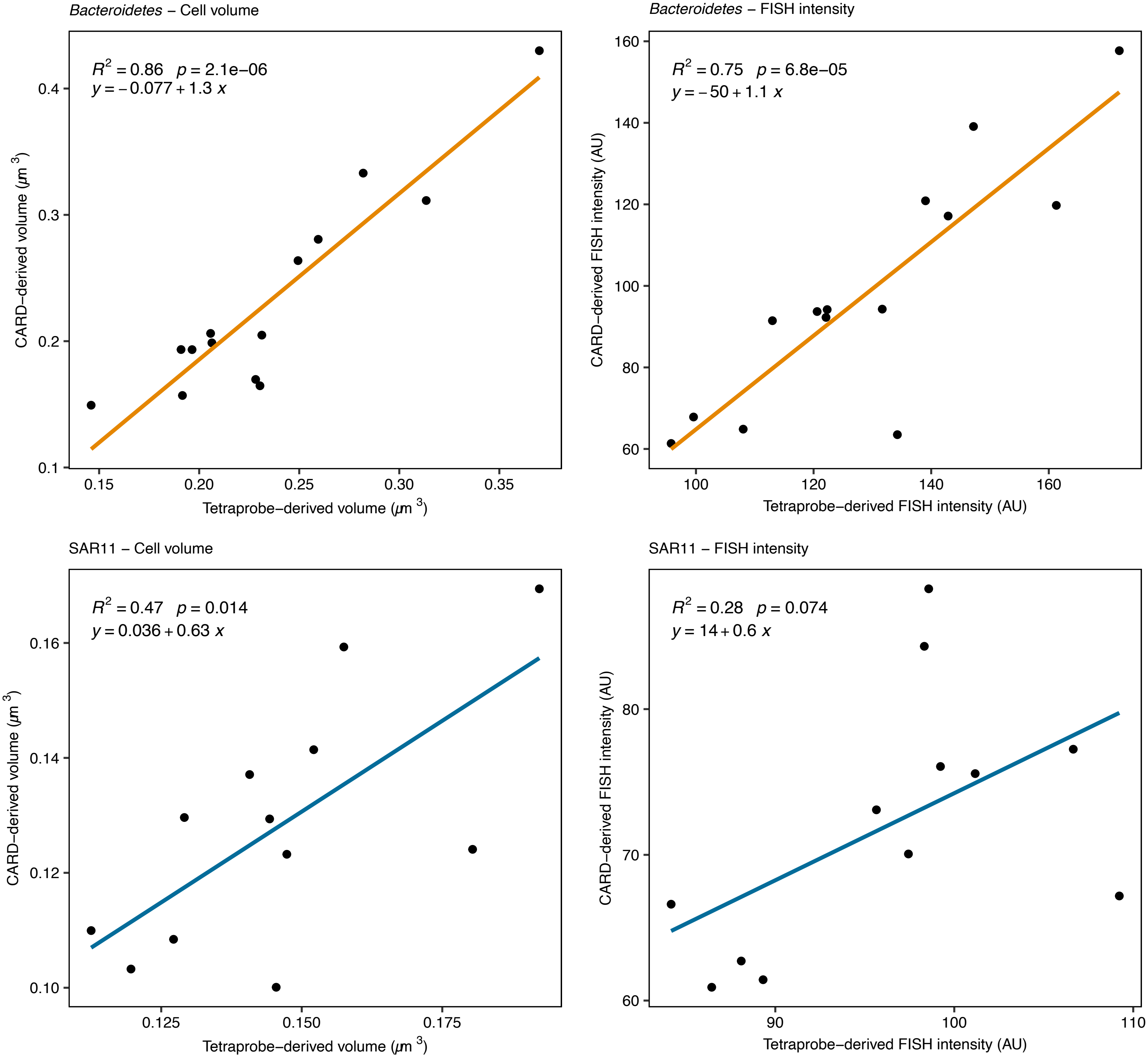

Figure S8 Correlation of CARD-FISH and tetra-labelled FISH cell volumes and signal intensities for *Bacteroidetes* and SAR11 cells from selected 2020 spring bloom dates. Displayed are means of each sampling day and linear regression of the means. The displayed statistics are for the linear regression model on the means.
