# Supplementary material for: In situ cell division and mortality rates of SAR11, SAR86, Bacteroidetes, and Aurantivirga during phytoplankton blooms reveal differences in population controls
Source: mSystems. 2023 May 17;8(3):e01287-22. doi: 10.1128/msystems.01287-22 (PMC10308942; doi:10.1128/msystems.01287-22)
Supplement: FIG S9 — Screenshots of the ACME tool. (A) is the image in the DAPI channel, (B) FISH channel, and (C) the autofluorescence channel. DAPI positive objects have a light-blue, FISH positive a green, and auto-fluorescent particles a red outline. Red box is a zoom-in on an example of a FISH positive cell with two local DAPI maxima (i.e., a dividing cell). Yellow circle is around an algae cell, yellow arrow points towards debris. Images are an example of 20th April and the samples were hybridized with the SAR11 mix. Each field of view is 1388x1040 pixel and each pixel has a height and width of 0.106 µm. [file msystems.01287-22-s0009.pdf]

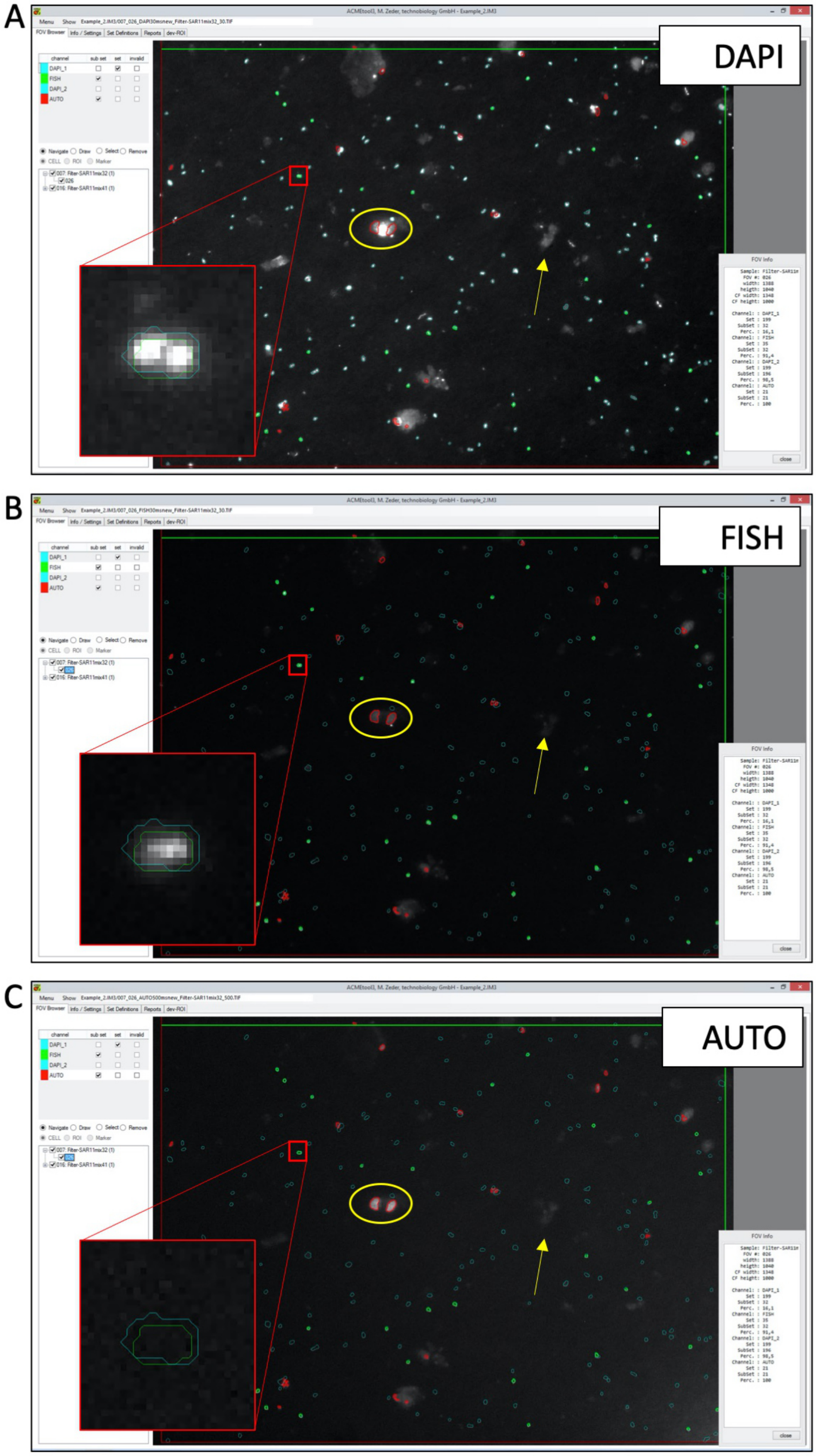

Figure S9 Screenshots of the ACME tool. (A) is the image in the DAPI channel, (B) FISH channel, and (C) the autofluorescence channel. DAPI positive objects have a light-blue, FISH positive a green, and auto-fluorescent particles a red outline. Red box is a zoom-in on an example of a FISH positive cell with two local DAPI maxima (i.e., a dividing cell). Yellow circle is around an algae cell, yellow arrow points towards debris. Images are an example of 20th April and the samples were hybridized with the SAR11 mix. Each field of view is 1388x1040 pixel and each pixel has a height and width of 0.106 μm.
