# Supplementary material for: In situ cell division and mortality rates of SAR11, SAR86, Bacteroidetes, and Aurantivirga during phytoplankton blooms reveal differences in population controls
Source: mSystems. 2023 May 17;8(3):e01287-22. doi: 10.1128/msystems.01287-22 (PMC10308942; doi:10.1128/msystems.01287-22)
Supplement: SUPPLEMENTAL FILE 1 — Supplementary Material and Methods and Results. [file msystems.01287-22-s0010.docx]

**Supplementary Information**

**Supplementary Material and Methods**

Catalyzed Reporter Deposition (CARD)-FISH

All mentioned chemicals were acquired by Carl Roth, Karlsruhe, Germany, if not stated otherwise. Microscopy filters were embedded in 0.1% LE agarose (w/v, Biozym, Hessisch Oldendorf, Germany) to reduce cell losses during sample handling. Cell walls were partly digested with 10 mg mL^-1^ lysozyme (Sigma Aldrich, Darmstadt, Germany) in lysozyme buffer (0.05 M EDTA, 0.1 M Tris-HCl pH 8.0) for 1 hour at 37°C. Endogenous peroxidases were inactivated with 0.15% H_2_O_2_ in methanol for 20 min at room temperature. Subsequently, samples were washed thoroughly in deionized water, air dried, and stored at -20°C until further processing.

CARD-FISH probes contained a horseradish peroxidase on their 5’ end for the signal amplification. FISH probes were diluted to a final concentration of 0.84 pmol mL^-1^ into the hybridization mixture (900 mM NaCl, 20 mM Tris-HCl pH 8.0, formamide (concentration depending on probe; see Table S2 at doi.org/10.6084/m9.figshare.22290166), 1% blocking reagent, 0.1 g mL^-1^ dextran sulfate, and 0.02% SDS). Humidity chambers were prepared by adding a tissue, soaked with a formamide-water mixture (concentration depending on probe), into an airtight household container. Sample filters were hybridized on petri dishes in the humidity chambers for 3 hours at 46°C. Thereafter, samples were washed first in washing buffer (20 mM Tris-HCl pH 8.0, 5 mM EDTA pH 8.0, 0.01% SDS, and NaCl (0.225 M, 0.159 M, or 0.08 M for 20%, 25%, or 35% formamide in hybridization mixture, respectively)) for 15 min at 48°C and afterwards in 1x PBS for 15 min at room temperature. For signal amplification, 1 µg mL^-1^ A488 tyramids (synthesized according to Pernthaler and Pernthaler (1) with Alexa 488 dyes (Thermo Fisher, Waltham, Massachusetts, USA)) were mixed with 0.0015% H_2_O_2_ in an amplification buffer (1x PBS, 2 M NaCl, 0.1% blocking reagent and 1 g mL^-1^ dextran sulfate). Filters were incubated in the amplification mixture in a humidity chamber (only water) for 45 min at 46°C. Subsequently, samples were thoroughly washed in deionized water and 96% ethanol, before DAPI staining and embedding in Citifluor:Vectashield (1:3).

Tetra-labelled (or ‘4-times labelled’) FISH

Tetra-labelled FISH probes were ordered from Biomers (Biomers, Ulm, Germnay) with Alexa 488 fluorophores conjugated to modified bases. The FISH hybridization protocol was shortened. No lysozyme digestion, deactivation of endogeneous peroxidases and CARD-amplification was applied. Experiments with tetra-labelled FISH probes were conducted on samples from 3^rd^, 10^th^, 17^th^, and 26^th^ March, 8^th^, 15^th^, 20^th^, 24^th^, 28^th^, and 29^th^ April, 11^th^ and 20^th^ May and done with probes targeting SAR11 and *Bacteroidetes*, as well as the negative control probe NON338 (results not shown).

ACME tool settings

Images were imported into the ACME tool. Field of views (FOVs) were manually approved based on images recorded in the DAPI channel. Unsuitable FOVs (e.g., out of focus, overexposure, off grid, etc.) were removed from the downstream analysis. High-quality images were first segmented within the ACME tool (kernel size = 19 pixel, offset = 11 pixel, removal of objects <21; Fig. S11). Subsequently, objects were identified based on the area of objects (DAPI channel: >24 pixel; FISH channel: >14.95 pixel), signal-to-background-ratio (DAPI channel: >2.7; FISH channel: >3.2), and circularity (DAPI channel: >0.55). Identified objects (DAPI positive) with a signal in the autofluorescence channel were neglected. Identified objects (DAPI positive) with a signal in the FISH channel were considered FISH-positive. In both cases, a threshold of a minimum overlap of 40% in signal area was chosen.

Cell concentrations were calculated in two different approaches. Cells stained with the SAR11-mix and CF319a FISH probe, relative amounts of DAPI-stained cells were calculated and extrapolated to total cell counts. We, thus, account for potential cell losses during the sample handling of the FISH protocol. Due to overall lower cell abundances, the SAR86 and AUR452 probes were hybridized on 10x filters (total volume: 100 ml). As these filters are more densly loaded, segmentation of individual DAPI-stained cells was not always possible. Hence, we directly calculated the concentrations based on the total number of FISH-stained cell per FOV.

MicrobeJ settings for frequency of dividing cell calculations

High quality images (see above) were additionally loaded in to the image processing software imageJ/Fiji (version 2.1.0/1.53e) (2) and were processed with the plug-in microbeJ (v 5.13l3). Object identification was done on the FISH channel in dark mode and an offset on the threshold value of -50. Objects had to be 7-250 pixel^2^, have a circularity of 0.55-max and a Z-score 2.8-max. Maxima were identified with the association “inside” (corresponding box was ticked), in dark and ‘Point’ mode with a tolerance of 25. Similar to the ACME tool, FISH-positive cells required to have a minimum of one DAPI signal and no autofluorescence signal.

Calculation of cell volume based on signal area

Volumes were calculated based on the geometrical approximation of a cylinder with hemispherical capping. In a two-dimensional projection, this results in a rectangle with two half-circles. The area *A* of the two-dimensional projections can be calculated with the radius *r* and the length *l* of the rectangle:

(1) $A=\pi r^{2}+2lr$ which can be reordered to (2) $l=\frac{A-\pi r^{2}}{2r}$ and the perimeter *P* (3) $P=2\pi r+2l$.

The area *A,* perimeter *P*, and circularity *C* of the two-dimensional microscopy images were exported from the ACME tool. Inserting (2) in (3) allows us to calculate *r* and *l*.

(4) $P=2\pi r+2(\frac{A-\pi r^{2}}{2r})$ |solving to r

(5) $r_{1,2}= \frac{P\pm\sqrt{P^{2}-4\pi A}}{2\pi}$

The Volume *V* can be calculated with the approximation of a cylinder with hemispherical caping $V=\frac{4}{3}\pi r^{3}+\pi r^{2}l$.

Metagenomic abundance estimates

The abundance of MAGs was determined as the quotient between the calculated truncated average sequencing depth (TAD80) (4) and the total sequencing depth of the microbial genomes (i.e., genome equivalence) (5). To determine TAD80 values for individual MAGs of each of the four groups analysed in this study, we performed competitive read mapping. First, we created a bowtie2 (6) database containing all contigs from the MAGs of each group. The mapping of reads to the concatenated file was performed using bowtie2 v. 2.4.2 (--no-discordant --no-mixed --reorder --no-unal). To obtain the individual mapping of each MAG, we filtered matching reads to each individual MAG and selected for at least 97% identity between the read and the MAG using the script ‘sam.filter.rb’ of the enveomics collection (7). Then, from the filtered mapping to each MAG, we determined the TAD80 value using the “BedGraph.tad.rb” script (-r 0.8) from the same script collection. Genome equivalents were determined using MicrobeCensus (5) for each metagenomic sample.

Taxonomic characterization of SAR11 MAGs

SAR11 single amplified genomes (SAGs), described in Haro-Moreno et al. (8), were retrieved from NCBI. Additionally, SAR11 isolate genomes described in Delmont et al. (9) were downloaded from <doi:10.6084/m9.figshare.5248945>. All genomes were aligned using GTDB-TK with <identify> and <align> (10). An approximately-maximum-likelihood phylogenetic tree was calculated using FastTree (11). The calculated tree was visualized with iTol (12), exported, and colourized with Affinity Designer (Serif, Nottingham, UK). Clade and sub-clade assignment are based on Haro-Moreno et al. (8) and Delmont et al. (9) (Fig. S12).

Growth Rate Index (GRiD)

GRiD values reported in the main text were retrieved using default settings of the GRiD software with a minimum coverage of 5. We expected some ambiguous read-mapping, especially for SAR11, due to genomic diversity and the difficulty to retrieve MAGs. We wanted to account for ambiguous read-mapping and to compare our results with different read-mappings. Besides running GRiD under default mode, we activated the -p flag, which enables the “reassignment of ambiguous reads using Pathoscope2”, according to the GRiD manual. Additionally, we used the mappings (sam files) generated from Bowtie2 (6) using the same settings described above for TAD80 determination.

Cell division rate calculations from Dilution experiments

Cell division rate estimates follow the first description of Landry and Hasset(13). Net growth rates can be calculated with time *t*, abundance at the start *N_0_*, abundance at the end point *N_t_*, and the respective dilution factor *d*:

$$Net growth=\left( \frac{1}{t} \right)*\ln\left( \frac{N_{t}}{N_{0}*d} \right)$$

The linear model of the apparent growth over the dilution factor can be used to estimate the grazing rate k (d^-1^; slope of the linear model) and cell division rate µ (d^-1^; y-axis intercept). The linear regressions were manually curated: In case of strong non-linear relation of cell abundances in dilution experiment (SAR11 and *Aurantivirga* 31^st^ March), undiluted samples were neglected. A non-linear dilution experiment indicates a saturation in predation and is a known bias to dilution experiments (14). More sophisticated models to deal with non-linear outcomes are discussed in Li et al. (14).

**Supplementary results**

Statistical evaluation of image cytometric data and cell division rates

The taxon-specific ribosome content was positively correlated to the respective cell volume (*R^2^*=0.94, *p*<2.2x10^-16^) and to the FDC (*R^2^*=0.29, *p*<2.2x10^-16^). The taxon-specific cell volume was positively correlated to the FDC (*R^2^*=0.27, *p*<2.2x10^-16^). The linear relation between FDC and the interaction term of cell division rate *µ* and used FISH probe (FDC ~ *µ**FISH probe) was significant (*µ* *p* < 0.0001; FISH probe *p* = 0.005; *µ*:FISH probe *p* = 0.019). A *post hoc* test with *p* value adjustment, following the tukey method, revealed that the y-axis (FDC) intercept is insignificantly different for all taxa (Table S8 at doi.org/10.6084/m9.figshare.22290166). The differences in the slopes for SAR86 and AUR452 were significant (*p* = 0.023) but insignificant in all other relations (but see CF319a – SAR86: *p* = 0.059; Table S9 at doi.org/10.6084/m9.figshare.22290166).

Metagenomic assessment of taxon-diversity

Four out of five assessed SAR11 MAGs were classified as SAR11 Cluster Ia.1 (Fig. S12). They were dominated by MAG r27, which contributed between 2.2% (19^th^ March 2020) up to 21.6% (11^th^ and 17^th^ May) to the entire microbial community. MAGs r30 (2.2 – 5.7%), r119 (2.1 – 5.0%), and r116 (≤1%) remained relatively stable throughout the spring bloom. Lower abundances (0.1 – 0.2%) were determined for MAG r31 belonging to the SAR11 cluster III (Fig. S12). The SAR86 community was dominated by MAG r157, which was least abundant on 26^th^ April (0.1%) and most abundant on 24^th^ May (3.5%). Additionally, MAG r159 contributed 0.6 – 0.7% between 19^th^ March and 5^th^ April, but contributed less for the rest of the sampling period. MAG r29 (0.2 – 0.5%) dominated the *Aurantivirga* community for most of the spring bloom, though r261 (max. 0.6%) and r179 (max. 0.4%) were most abundant beginning and mid of May, respectively (Fig. S7).

For *Bacteroidetes*, first, MAGs belonging to GTDB-Tk genus-level clade MAG-121220-bin8 were most abundant until mid-April (Fig. S8, red bar) with MAGs r79, r45, and r152 contributing 2.0 – 4.0% until 10^th^ April (Fig. S8). In May, MAGs of the genus-level clade *Ulvibacter_B* dominated the *Bacteroidetes* community (Fig. S8, yellow bar). The clade was highly diverse with individual MAGs r111, r126, r136, r154, r187, r216, r255, r276, and r78 accounting <1% each to the entire bacterial community (Fig. S8).

**Bibliography**

1. Pernthaler A, Pernthaler J. 2007. Fluorescence in situ hybridization for the identification of environmental microbes, p 153-164, Protocols for nucleic acid analysis by nonradioactive probes. Springer.

2. Schindelin J, Arganda-Carreras I, Frise E, Kaynig V, Longair M, Pietzsch T, Preibisch S, Rueden C, Saalfeld S, Schmid B. 2012. Fiji: an open-source platform for biological-image analysis. Nature methods 9:676-682.

3. Ducret A, Quardokus EM, Brun YV. 2016. MicrobeJ, a tool for high throughput bacterial cell detection and quantitative analysis. Nature microbiology 1:1-7.

4. Orellana LH, Francis TB, Ferraro M, Hehemann J-H, Fuchs BM, Amann RI. 2021. *Verrucomicrobiota* are specialist consumers of sulfated methyl pentoses during diatom blooms. The ISME Journal:1-12.

5. Nayfach S, Pollard KS. 2015. Average genome size estimation improves comparative metagenomics and sheds light on the functional ecology of the human microbiome. Genome biology 16:1-18.

6. Langmead B, Salzberg SL. 2012. Fast gapped-read alignment with Bowtie 2. Nature methods 9:357-359.

7. Rodriguez-R LM, Konstantinidis KT. 2016. The enveomics collection: a toolbox for specialized analyses of microbial genomes and metagenomes. PeerJ Preprints,

8. Haro‐Moreno JM, Rodriguez‐Valera F, Rosselli R, Martinez‐Hernandez F, Roda‐Garcia JJ, Gomez ML, Fornas O, Martinez‐Garcia M, López‐Pérez M. 2020. Ecogenomics of the SAR11 clade. Environmental Microbiology 22:1748-1763.

9. Delmont TO, Kiefl E, Kilinc O, Esen OC, Uysal I, Rappe MS, Giovannoni S, Eren AM. 2019. Single-amino acid variants reveal evolutionary processes that shape the biogeography of a global SAR11 subclade. eLife 8:e46497.

10. Chaumeil P-A, Mussig AJ, Hugenholtz P, Parks DH. 2020. GTDB-Tk: a toolkit to classify genomes with the Genome Taxonomy Database. Bioinformatics 36:1925-1927.

11. Price MN, Dehal PS, Arkin AP. 2010. FastTree 2–approximately maximum-likelihood trees for large alignments. PloS one 5:e9490.

12. Letunic I, Bork P. 2021. Interactive Tree Of Life (iTOL) v5: an online tool for phylogenetic tree display and annotation. Nucleic acids research 49:W293-W296.

13. Landry M, Hassett R. 1982. Estimating the grazing impact of marine micro-zooplankton. Marine biology 67:283-288.

14. Li QP, Franks PJ, Landry MR. 2017. Recovering growth and grazing rates from nonlinear dilution experiments. Limnology and Oceanography 62:1825-1835.
